# Regulation of Nutritional Metabolism in Transition Dairy Cows: Energy Homeostasis and Health in Response to Post-Ruminal Choline and Methionine

**DOI:** 10.1371/journal.pone.0160659

**Published:** 2016-08-08

**Authors:** Feifei Sun, Yangchun Cao, Chuanjiang Cai, Shengxiang Li, Chao Yu, Junhu Yao

**Affiliations:** College of Animal Science and Technology, Northwest A&F University, Yangling, Shaanxi, 712100, P. R. China; University of Florida, UNITED STATES

## Abstract

This study investigated the effects of rumen-protected methionine (RPM) and rumen-protected choline (RPC) on energy balance, postpartum lactation performance, antioxidant capacity and immune response in transition dairy cows. Forty-eight multiparous transition cows were matched and divided into four groups: control, 15 g/d RPC, 15 g/d RPM or 15 g/d RPC + 15 g/d RPM. Diet samples were collected daily before feeding, and blood samples were collected weekly from the jugular vein before morning feeding from 21 days prepartum to 21 days postpartum. Postpartum dry matter intake (DMI) was increased by both additives (*P* < 0.05), and energy balance values in supplemented cows were improved after parturition (*P* < 0.05). Both RPC and RPM decreased the plasma concentrations of non-esterified fatty acids (NEFA), β-hydroxybutyric acid (BHBA), total cholesterol (TC) and low-density lipoprotein cholesterol (LDL-C) (*P* < 0.05), but increased the plasma levels of glucose, very-low-density lipoprotein (VLDL) and apolipoprotein B100 (ApoB 100, *P* < 0.05). The supplements improved milk production (*P* < 0.05), and increased (*P* < 0.05) or tended to increase (0.05 < *P* < 0.10) the contents of milk fat and protein. The post-ruminal choline and methionine elevated the blood antioxidant status, as indicated by total antioxidant capacity (T-AOC), glutathione peroxidase (GSH-Px) activity and the vitamin E concentration (*P* < 0.05), and reduced the plasma malondialdehyde (MDA) level (*P* < 0.05). Furthermore, RPM and RPC elevated the plasma interleukin 2 (IL-2) concentration and the CD4^+^/CD8^+^ T lymphocyte ratio in peripheral blood (*P* < 0.05). Alternatively, the levels of tumor necrosis factor-α (TNF-α) and IL-6 were decreased by RPM and RPC (*P* < 0.05). Overall, the regulatory responses of RPC and RPM were highly correlated with time and were more effective in the postpartum cows. The results demonstrated that dietary supplementation with RPC and RPM promoted energy balance by increasing postpartal DMI and regulating hepatic lipid metabolism, improved postpartum lactation performance and enhanced antioxidant capacity and immune function of transition dairy cows.

## Introduction

The transition period spans from three weeks before to three weeks after calving. During this period, dairy cows are more susceptible to metabolic disorders, such as fatty liver, ketosis, retained placenta, hypocalcemia and clinical mastitis [1,2]. Due to the rapid growth of the fetus and the postnatal onset of lactation, the nutrient requirements of cows over this period are substantially increased. However, dietary energy intake does not meet this nutrient demand, thereby resulting in a negative energy balance (**NEB**) [3,4]. Subsequently, NEB triggers body fat mobilization to meet the increasing energy demands of the cows. As a consequence of fat mobilization, higher levels of non-esterified fatty acids (**NEFA**) are generated in adipose tissue and released into the blood circulation [5,6]. An elevated NEFA concentration is a typical hallmark of transition cows, and the insufficient metabolic capability of hepatic NEFA has been demonstrated to adversely affect animal health and to impair lactation performance as well as subsequent reproductive performance [7,8]. Thus, nutritional strategies to reduce the adverse effects of NEFA, such as mitigating the body fat mobilization and accelerating the removal of hepatic of NEFA, is urgently required to safeguard the health of transition dairy cows, and many researchers have focused their attention on this area.

Circulatory NEFA can be either utilized by the mammary gland to synthesize milk fat or transported to the liver for further metabolism. The liver plays a crucial role in the homeostasis of NEFA [5]. There are three primary pathways that metabolize NEFA in the liver: (1) complete oxidation to generate ATP in hepatic mitochondria or peroxisomes, (2) incomplete oxidation to produce ketone bodies, including acetone, acetoacetate, and β-hydroxybutyric acid (**BHBA**), or (3) re-esterification to form triglycerides (**TG**). The TG can either accumulate in hepatocytes or be transported out of the liver in the form of very-low-density lipoprotein (**VLDL**) [9–11]. However, the ability to completely oxidize NEFA and synthesize VLDL is limited, thereby increasing the incidence rates of ketosis and fatty liver [5, 12].

Choline and methionine serve as pivotal nutrients involved in the transport of hepatic lipids by promoting the synthesis of phosphatidylcholine to package VLDL. Specifically, both of them play vital roles in 1-carbon units transfer of dairy cows, modulate the synthesis of S-adenosylmethionine (SAM) in methionine cycle, and SAM functions as the most important methyl donor in the phosphatidylethanolamine to phosphatidylcholine formation [13]. In addition, deficiency in choline and methionine has been reported to exacerbate hepatic lipid infiltration [14,15]. Therefore, these nutrients have been considered as essential molecules for transition dairy cows. Supplementation with rumen-protected choline (**RPC**) has been reported to decrease liver TG deposition [16,17], the plasma BHBA level [16] and the blood NEFA concentration [16,18], enhance energy balance and improve liver health in transition dairy cows. However, inconsistent with the above results, Ardalan et al. [14] found no effect of RPC supplementation (14.4 g/d per cow, calculated as choline chloride) on these characteristics in either prepartum or postpartum dairy cows. Moreover, methionine is considered as a limiting amino acid for milk performance, and rumen-protected methionine (**RPM**) is typically added to the diet of dairy cows to facilitate milk protein production. Although the regulatory effect of RPM on lipid metabolism and energy homeostasis in transition cows has received some attention, investigations are comparatively less, and existed conclusions are inconsistent. In addition, the potential interactions between RPC and RPM have rarely been studied.

Furthermore, the physiological challenge of fat mobilization can lead to excessive hepatic lipid peroxidation and produces numerous free radicals, which are beyond the scavenging capacity of antioxidant system, thereby disrupting the redox balance [4,13], resulting in oxidative stress and decreased immune function [19,20]. Choline and methionine have been shown to alleviate oxidative stress as well as enhance immunity in rodents, humans and fish [21–23]. Nevertheless, whether RPC and RPM affect the antioxidant status and immunologic function of transition dairy cows remains not entirely clear. Osorio et al. [13] demonstrated that dietary RPM supplementation could expedite the synthesis of glutathione, an important antioxidant peptide, and improve the antioxidant capacity of transition cows. In fact, decreased circulating BHBA and NEFA levels could indirectly mitigate the damage of these adverse fatty acids to the antioxidant system and lymphocytes [1,12].

Hence, we hypothesized that post-ruminal availability of choline, methionine or both could alleviate NEB and improve health status of transition dairy cows. The objectives of the present investigation were 1) to study the effects of dietary supplementation of RPM and/or RPC on energy balance, lipid metabolism and postpartum lactation performance and 2) to determine potential regulatory effects of RPM and RPC on antioxidant capacity and immune function.

## Materials and Methods

### Ethics statement

The use of animals and the performance of all experimental protocols were approved by the Northwest A&F University Animal Welfare Committee.

### Animals, diet, and experimental design

The animal feeding experiment was conducted at the New Continent Dairy Farm (Yangling, Shaanxi Province, China). Forty-eight healthy multiparous transition Chinese Holstein dairy cows were matched according to their body condition score, expected day of calving and milk yield during the previous lactation cycle and were divided into four groups of twelve cows per group. Each group was assigned to one of the following four treatments in a 2×2 factorial design: control (basal diet, **T**_**0**_), 15.0 g/d RPC (**T**_**C**_), 15.0 g/d RPM (**T**_**M**_), or 15.0 g/d RPC + 15.0 g/d RPM (**T**_**CM**_). RPC was supplied as 60.0 g/d ReaShure^®^ (choline chloride content 25.0%, rumen bypass rate 85.0%, Balchem Corp., New Hampton, USA) [24]. RPM was provided as 17.7 g/d Mepron^®^ (DL-methionine content 85.0%, rumen bypass rate 80.0%, Evonik Industries, Mobile, Alabama, USA) [25].

Two different diets, prepared as total-mixed ration (**TMR**), were used for the pregnancy and lactation periods. In addition, to avoid the possible alterations of rumen environment because of diet changes, both far-off and close-up cows were fed the same TMR during the dry period. The ingredients of each TMR are listed in Table 1. The diet provided during the pregnancy period contained 11.92% CP and 1.50 Mcal NE_L_ (dry matter basis), and the diet provided during the lactation period contained 15.02% CP and 1.67 NE_L_ per kg dry matter. Cows were fed three times per day (05:30, 13:30 and 19:30) and free access to water. For supplementation of RPC and RPM, non-supplementation, 60.0 g/d ReaShure^®^, 17.7 g/d Mepron^®^ or 60.0 g/d ReaShure^®^ + 17.7 g/d Mepron^®^ were mixed with 25.0 g corn meal and 50.0 g wheat bran to form four basal mixtures. To balance the bioavailable nutrients in the coated materials, 15.0 g vegetable oil for non-RPC groups as well as 0.18 g vegetable oil plus 0.53 g microcrystalline cellulose for non-RPM groups were added to constitute four total mixtures. The mixtures were offered to cows three times a day before feeding the basal TMR. The whole animal experimental period lasted for 42 days, 21 days before and after calving, respectively.

**Table 1:** Ingredients and chemical composition of experimental diets (total-mixed ration, dry matter basis)^1^.

Items	Prepartum	Postpartum
Ingredients, %		
Corn silage	41.94	28.19
Alfalfa hay	0	10.25
Wheat straw	23.48	10.25
Corn grain	17.28	24.91
Wheat bran	4.86	8.71
Soybean meal	5.04	7.79
Cottonseed meal	5.28	7.18
Limestone	0.66	1.33
Sodium bicarbonate	0.38	0.50
Calcium phosphate	0.34	0.12
Salt	0.23	0.30
Magnesium oxide	0.17	0.15
Vitamin and mineral premix^2^	0.34	0.32
Chemical composition, %		
CP	11.92	15.02
EE	3.77	4.40
Starch	26.48	29.13
Ash	6.81	5.27
NDF	45.32	36.04
ADF	27.32	21.97
Ca	0.51	o.78
P	0.45	0.45
Lys: Met^3^	3.13: 1	3.15: 1
NE_L_, Mcal/kg dry matter^3^	1.51	1.69

^1^ CP = crude protein, EE = ether extracts, NDF = neutral detergent fiber, ADF = acid detergent fiber, Ca = calcium, P = phosphorus, Lys: Met = the ratio of lysine to methionine, and NE_L_ = net energy for lactation.

^2^ Vitamin and mineral premix (per kilogram of total-mixed ration, dry matter basis): 10.5 mg Cu, 9.80 mg Zn, 12.00 mg Mn, 0.11 mg Co, 0.32 mg I, 0.15 mg Se, 2,500 IU vitamin A, 500 IU vitamin D_3_, and 40 IU vitamin E for pregnant cows; 6.4 mg Cu, 12.80 mg Zn, 15.20 mg Mn, 0.09 mg Co, 0.60 mg I, 0.17 mg Se, 2,500 IU vitamin A, 500 IU vitamin D_3_, and 70 IU vitamin E for lactating cows.

^3^ Lys: Met and NE_L_ were calculated and estimated using Cornell-Penn-Miner Dairy (CPM-Dairy, version 3.0.8.1) software according to the NRC 2001 model.

### Sample collection and pretreatment

The TMR samples were collected daily before feeding, and stored at -20°C. At the end of experiment, the samples were pooled, subsampled and used for further chemical analysis. The amounts of TMR offered and residual were recorded daily for each cow to calculate feed intake. Blood samples were collected into an evacuated heparin-coated tube (Kangjian Co., Ltd, Jiangsu, China) from the jugular vein before morning feeding on days -21, -14, -7, 0 (calving day), 7, 14 and 21. Blood samples were placed into ice immediately, and transferred to the lab within an hour. An aliquot of blood samples was centrifuged at 3000 rpm for 10 min. The supernatant was stored at -80°C for further analysis. The rest of blood samples were used to isolate lymphocytes to measure T-lymphocyte subsets. Cows were milked three times per day (05:00, 13:00 and 19:00), and milk samples were immediately transferred back to the lab at 4°C for milk composition determination via an automatic milk composition analyzer (FOSS, Denmark). The milk yield was calculated as 4% fat-corrected milk (FCM) using an equation: FCM (kg) = 0.4 M + 15 F, where M meant the mass of milk production (kg), and F meant the milk fat production (kg).

### Chemical analysis of TMR

The dry matter content of each ration was detected after drying at 135°C for 3 h, and the ash content was detected following combustion at 550°C for 6 h according to the protocols of the AOAC [26]. The Kjeldahl method [27] was used to determine the levels of crude protein (**CP**, 6.25×N) using an automated nitrogen analyzer (FOSS, DK-3400 Hillerød, Denmark). The total starch content in the TMR was determined via an enzymatic method (α-amylase and amyloglucosidase) using a commercial kit (Megazyme, Megazyme International Ireland Ltd., Bray, Ireland). To analyze the neutral detergent fiber (**NDF**) and acid detergent fiber (**ADF**) contents, heat-stable α-amylase (Sigma A3306; Sigma–Aldrich, Shanghai, China) and sodium sulfite were used according to the methods previously established by Van Soest et al. [27]. The total calcium (**Ca**) and phosphorus (**P**) levels were determined according to the National Measurement Principles of China (GB/T 5009.92–2003 and GB/T 5009.87–2003, respectively). The dietary lysine to methionine ratio (**Lys: Met**) and the net energy for lactation (**NE**_**L**_**)** of each ration were estimated based on the included ingredients using the Cornell-Penn-Miner Dairy (CPM-Dairy, version 3.0.8.1) software.

### Analyses of plasma metabolites

The frozen plasma samples were thawed in an electrically heated thermostatic water bath (Jinghong, Shanghai, China) at 37°C for 15 min and were then used for various analyses. Spectrophotometric methods were used to determine the plasma levels of total protein (**TP**), albumin (**ALB**), glucose, total bilirubin (**TBIL**), glutamate pyruvate transaminase (**GPT**), glutamic oxalacetic transaminase (**GOT**), alkaline phosphatase (**ALP**), blood urea nitrogen (**BUN**) and NEFA using commercial kits purchased from Jiancheng Bioengineering Institute (Nanjing, China). The inter coefficients of variation (CVs) were 6.70%, 8.27%, 4.29%, 4.66%, 5.42%, 6.63%, 7.08% and 9.79%, respectively. All procedures were conducted in accordance with the manufacturers’ user manuals. The absorbance at specific wavelengths was recorded using a microplate reader (Power Wave XS2, BioTek, USA).

The Total Cholesterol (**TC**) Assay Kit was purchased from Cell Biolabs Inc. (San Diego, USA), and the Triglyceride Reagent Kit was obtained from BHKT Reagent Co., Ltd. (Beijing, China); both of these assays are based on an enzymatic colorimetric method. The plasma low-density lipoprotein cholesterol (**LDL-C**) measurement was conducted using a Low-density Lipoprotein Cholesterol Reagent Kit (BHKT Reagent Co., Ltd., Beijing, China) based on the selective precipitation procedure [28]. The high-density lipoprotein cholesterol (**HDL-C**) content was determined using a High-density Lipoprotein Cholesterol Reagent Kit (Dongou Diagnostic Products Co., Ltd., Wenzhou, China), which was based on a phosphotungstic acid magnesium chloride (PTA-Mg^2+^) precipitation protocol as described by Lopes et al. [29]. The inter-CVs of TC, LDL-C and HDL-C were 3.23%, 8.24% and 6.37%, respectively.

The enzyme-linked immunosorbent assay (**ELISA**) method was used to determine the plasma VLDL, apolipoprotein B100 (**ApoB 100**) and BHBA levels using three commercial kits from Cloud-Clone Corporation (Houston, USA): the Bovine Very Low-density Lipoprotein ELISA Kit, the ApoB 100 ELISA Kit and the BHBA ELISA Kit, respectively. The inter and intra CVs were 8.83% and 2.58%. Briefly, a blank, a series of seven standards, and samples, each 100 μL, were incubated for 2 h at 37°C. The solution was discharged (no washing applied). Then, 100 μL of Detection Reagent A was added, and the solution incubated for 1 h at 37°C. Next, the plate was washed three times with 350 μL of 1×Wash Solution. Afterwards, 100 μL of Detection Reagent B was added, and the solution was incubated for 30 min at 37°C. The plate was washed five times. Finally, 90 μL of Substrate Solution was added, and the solution was incubated for 20 min at 37°C in dark, followed by the addition of 50 μL of Stop Solution and agitation. The optical density at 450 nm was recorded using a microplate reader (Power Wave XS2, BioTek, USA).

### Plasma antioxidant status

In plasma samples, the total antioxidant capacity (**T-AOC**) were analyzed using a ferric reducing antioxidant power (FRAP) method described by Wang et al. [30], and activities of glutathione peroxidase (**GSH-Px**) and superoxide dismutase (**SOD**), as well as the malondialdehyde (**MDA**) concentration, were measured following the methods of Zhang et al. [31]. The catalase (**CAT**) activity was determined using a commercial kit (Jiancheng Bioengineering Institute Nanjing, China), based on the decomposition of hydrogen peroxide. A colorimetric method was used to detect plasma vitamin E content via an assay kit (Jiancheng Bioengineering Institute Nanjing, China). Briefly, 0.1 mL sample was added into an extraction buffer, mixed for 1 min in a vortex oscillation blender (Qilinbeier, Jiangsu, China), then centrifuged at 3500 rpm for 10 min. After that, 0.8 mL upper supernatant was taken to conduct a chromogenic reaction. Finally, the absorbance at 533 nm was recorded by a microplate reader (Power Wave XS2, BioTek, USA). The inter CVs of T-AOC, GSH-Px, SOD, MDA, CAT and vitamin E were 3.92%, 4.55%, 8.04%, 3.17%, 6.28% and 2.44%, respectively.

### Immune function

According to a method previously reported by Gao et al. [32], a radioimmunoprecipitation assay (**RIA**) was applied to determine the plasma levels of three interleukins (**IL-2**, **IL-4** and **IL-6**) using RIA kits (Puerweiye Biological Technology, Beijing, China). The plasma tumor necrosis factor-α (**TNF-α**) concentration was assessed using a Bovine TNF-α ELISA Kit (Jiancheng Bioengineering Institute, Nanjing, China). Firstly, a standard quadratic curve was made using the given standard substance (R^2^ = 0.9992). The operating steps was the same as mentioned above. The optical density at 450 nm was read via a microplate reader (Power Wave XS2, BioTek, USA). At last, the TNF-α concentration was calculated according to the established standard curve. The inter CVs of IL-2, IL-4, IL-6 and TNF-α were 6.30%, 4.22%, 7.58% and 8.03%, respectively.

In peripheral blood samples, the quantities of T-lymphocyte subtypes, particularly the **CD4**^**+**^**/CD8**^**+**^ ratio, were analyzed via a flow cytometric method. Briefly, 1 mL of heparin-anticoagulant blood was pipetted into a tube, 1 mL of phosphate buffered saline (**PBS**) precooled to 4°C was added, and the solution was mixed gently with a pipette (Eppendorf, Hamburger, Germany). Then, the mixture was transferred to a centrifugal tube (pre-placed at room temperature), and 2 mL of bovine peripheral lymphocyte separation medium (TBD Science, Tianjin, China) was added slowly and carefully along the tube wall, followed by centrifugation at 2000 rpm at 20°C for 25 min. Consequently, the liquid was separated into three phases. The lymphocytes in the middle milky layer was carefully pipetted into an ultra-clean centrifugal tube precooled to 4°C, mixed slowly with 2 mL of PBS, and centrifuged again at 2000 rpm at 4°C for 15 min. After removing the supernatant, 2 mL of PBS was added, and the solution was centrifuged at 2000 rpm at 4°C for 10 min. The supernatant was discarded, and the precipitated lymphocytes was re-suspended in 500 μL of PBS. Then, the cell number was counted under an inverted microscope (Nikon, Tokyo, Japan). Following this procedure, 1.0×10^6^ cells were pipetted into a flow cytometry tube (Becton, Dickinson and Company, New Jersey, USA). Three monoclonal antibodies, 10 μL of Rat Anti-Bovine CD3:FITC (GeneTex Inc., Southern California, USA), 5 μL of Mouse Anti-Bovine CD4:APC (AbD Serotec, Oxford, UK), and 10 μL of Mouse Anti-Bovine CD8:PE (AbD Serotec, Oxford, UK), and 0.5 mL of PBS were sequentially added. Then, the solution was mixed and incubated in the dark at 4°C for 30 min. Finally, the mixture was analyzed using a flow cytometer (FACSAria III, Becton, Dickinson and Company, New Jersey, USA) to quantify T-lymphocyte subtypes.

### Statistical analysis

The effects of supplementation with RPC and/or RPM on all examined parameters were statistically analyzed using the MIXED procedure of Statistical Analysis System 9.2 software (SAS Institute Inc., 2007) using the following model [33]:

$${\text{Y}}_{\text{ijkl}}=\mu +\alpha_{\text{i}}+\beta_{\text{j}}+\gamma_{\text{ij}}+{\text{w}}_{\text{k}}+\varepsilon_{\text{ijkl}}$$

where Y_ijkl_ is the dependent variable; μ is the overall mean; α_i_ is the effect of the *i*th RPC treatment, β_j_ is the effect of the *j*th RPM treatment; γ_ij_ is the interaction effect between the *i*th RPC treatment and the *j*th RPM treatment (RPC_i_×RPM_j_); ω_k_ is the effect of the *k*th week (day); and ε_ijkl_ is the random experimental error of the *l*th animal at the *i*th RPC treatment, the *j*th RPM treatment and the *k*th week (or day).

The REPEATED procedure was used for variables repeatedly measured over time, such as the plasma glucose concentration, which was analyzed throughout the experimental period. The interactive effects between treatments and time (C × Time, M × Time and C ×M × Time) could be obtained via this Repeated Measurement Model. Moreover, one-way ANOVA was conducted to compare the differences between the four treatment groups over the entire period or at a given time point. We used Data Processing System 7.05 (DPS 7.05, Zhejiang University, China) to reject abnormal data, which was based on 3σ, Dixon and Grubbs Criterion. To explain specially, the TMR composition was different during the prepartum and postpartum period, and we still fed prepartum diet to cows to avoid two different diets in the calving day. For instance, if a cow gave birth to a calf at 16:00, the prepartum diet was still given at the evening feeding, and the lactation diet was given the next morning. Although a same diet was given during the prepartum period and calving day, the intakes in calving day decreased a lot, thus we presented the intakes as prepartum, calving and postpartum, respectively. A significant difference was declared at *P* < 0.05, and 0.05 < *P* ≤ 0.10 was considered to indicate a tendency.

## Results

### Dietary intake and energy balance

**Fig 1:**
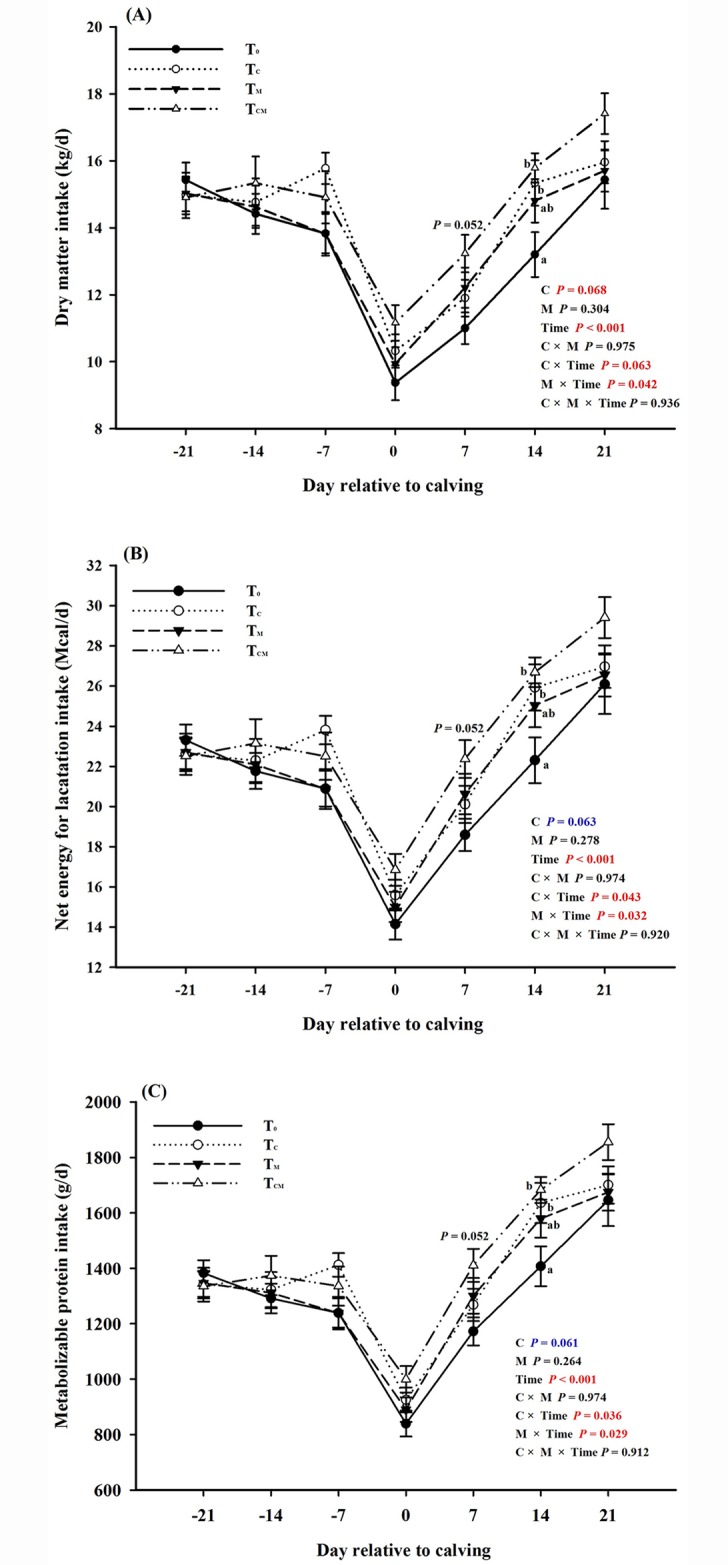
Dynamic effects of dietary RPC and RPM supplementation on the intakes of dry matter, net energy for lactation and metabolizable protein in transition dairy cows. ^a, b^ Values with different superscript letters at the same time point are significantly different (*P* < 0.05).

The weekly changes of the intakes of dry mater (DM), net energy for lactation (NE_L_) and metabolizable protein (MP) over the entire experimental period were shown in Fig 1. Over the prepartum period, intakes of DM, NE_L_ and MP declined continuously except for a slight increase in the T_C_ group at -7 d. A sharp decline of these parameters was observed in all four groups on calving day. Over the three weeks of the postpartum period, intakes progressively increased over time in all animals, although intakes in the T_C_ and T_CM_ groups was significantly greater than that in the control and T_M_ groups (*P* < 0.05). Moreover, the intakes at week 2 of the postpartum period tended to be higher in the T_M_ group than in the T_0_ group (*P* = 0.052). Depending on the supplements included, the average intakes of DM, NE_L_ and MP during the postpartal period was 1.0–2.3 kg/d higher in the groups provided with a supplemented diet (*P* < 0.05) than in the control group (Table 2). Supplementation of choline increased the intakes during the calving day (main effect *P* < 0.05). Over the whole period, choline tended to increase the intakes of DM, NE_L_ and MP (*P* = 0.068, 0.063 and 0.061, respectively), and all of the intakes were significantly affected by weeks (Time *P* < 0.001). Besides, the interactions between supplements and time were significant among all the three intakes (interaction *P* < 0.05).

**Table 2:** Effects of supplementation with rumen-protected choline (T_C_), rumen-protected methionine (T_M_) or both (T_CM_) on dry matter intake and blood parameters associated with energy balance during different periods of transition dairy cows^1^.

Items	T_0_	T_C_	T_M_	T_CM_	SEM	*P*-value		
						C	M	C×M
Prepartum								
DMI, kg/d	14.56	15.17	14.50	15.05	0.589	0.328	0.877	0.961
NEFA, mmol/L	0.89	0.87	0.89	0.86	0.031	0.446	0.981	0.815
BHBA, mmol/L	0.61	0.62	0.61	0.61	0.010	0.407	0.673	0.576
Glucose, mmol/L	3.65	3.79	3.67	3.77	0.037	0.002	0.988	0.751
Calving day								
DMI, kg/d	9.37	10.33	9.94	11.16	0.512	0.040	0.181	0.800
NEFA, mmol/L	1.40	1.33	1.35	1.34	0.091	0.704	0.865	0.764
BHBA, mmol/L	1.08	1.17	1.07	1.02	0.038	0.642	0.043	0.087
Glucose, mmol/L	4.10	4.48	4.38	4.94	0.110	<0.001	0.002	0.427
Postpartum								
DMI, kg/d	13.21	14.40	14.25	15.48	0.516	0.025	0.048	0.967
NEFA, mmol/L	1.24	0.97	1.06	0.90	0.021	<0.001	<0.001	0.027
BHBA, mmol/L	1.02	0.92	0.94	0.86	0.009	<0.001	<0.001	0.301
Glucose, mmol/L	3.61	3.73	3.71	3.80	0.026	<0.001	0.002	0.568

^1^DMI = dry matter intake, NEFA = non-esterified fatty acids, BHBA = β- hydroxybutyric acid. Prepartum refers to the mean values of days 21, 14, and 7 before the calving day, and postpartum represents the mean values of days 7, 14, and 21 after calving day.

**Fig 2:**
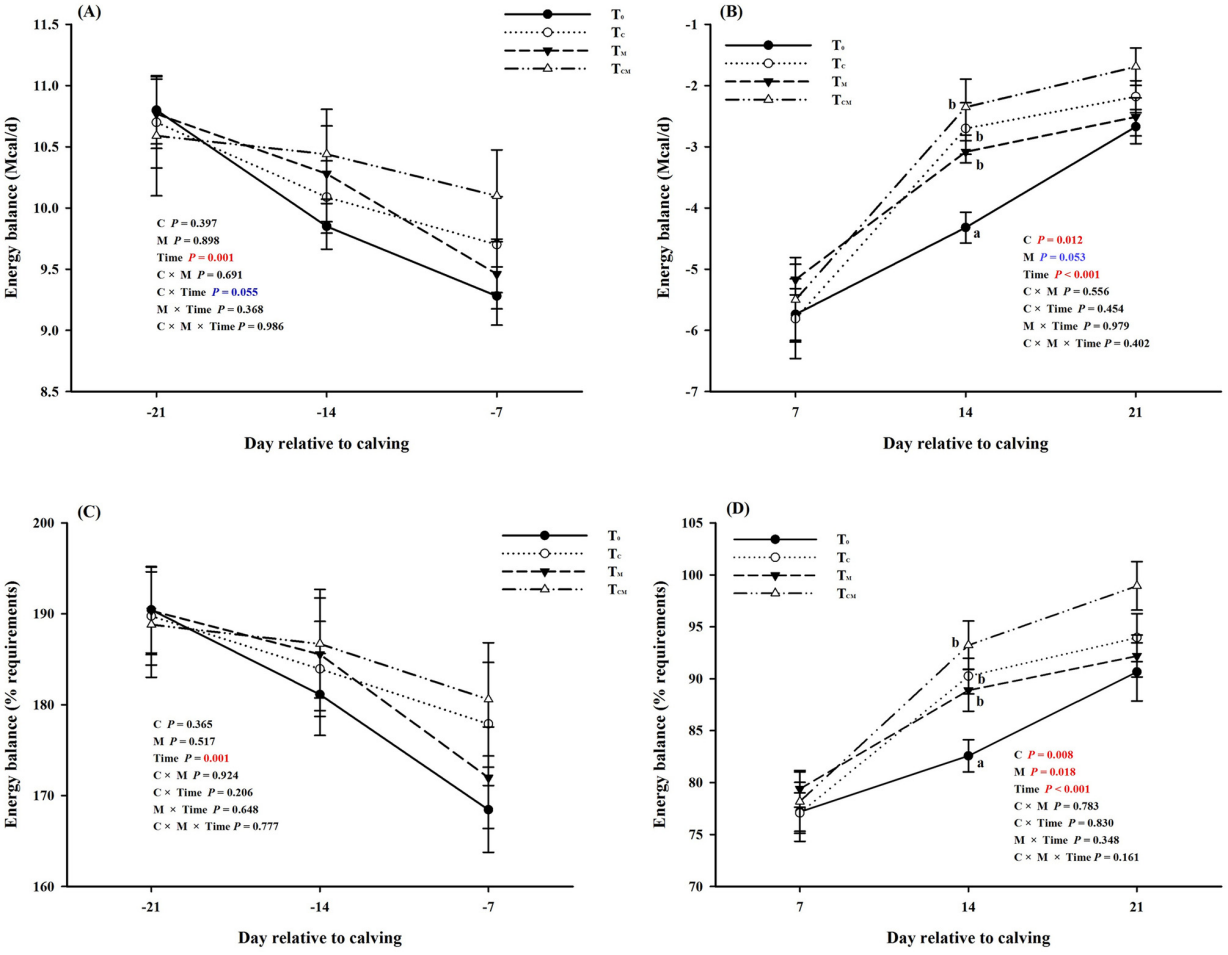
Prepartal and postpartal energy balance (as Mcal/d or %requirements) of transition dairy cows in response to supplementation of rumen-protected choline (T_C_), rumen-protected methionine (T_M_) or both (T_CM_). ^a, b^ Values with different superscript letters at the same time point are significantly different (*P* < 0.05).

The energy balance values of tested cows were calculated according to NRC (2001) and presented in Fig 2. With the approaching of the parturition (from -21 d to -7 d), the energy balance decreased obviously, but values were still above zero. Neither RPC nor RPM influenced energy balance of prepartal cows (main effect *P* > 0.05). During the postpartum period, all the cows were in a state of NEB, and the energy balance values increased from 7 d to 21 d. The RPC increased postpartum energy balance (main effect *P* < 0.05), and the RPM increased (as % requirements, *P* < 0.05) or tended to increase (as Mcal/d, *P* = 0.053) the energy balance. Whether it was before or after parturition, the energy balance was highly correlated with weeks (time *P* < 0.001). In particular, the energy balance in treatment groups was improved at 14 d when compared to the T_0_ group.

### Levels of lipid mobilization indicators and lipids in blood

**Fig 3:**
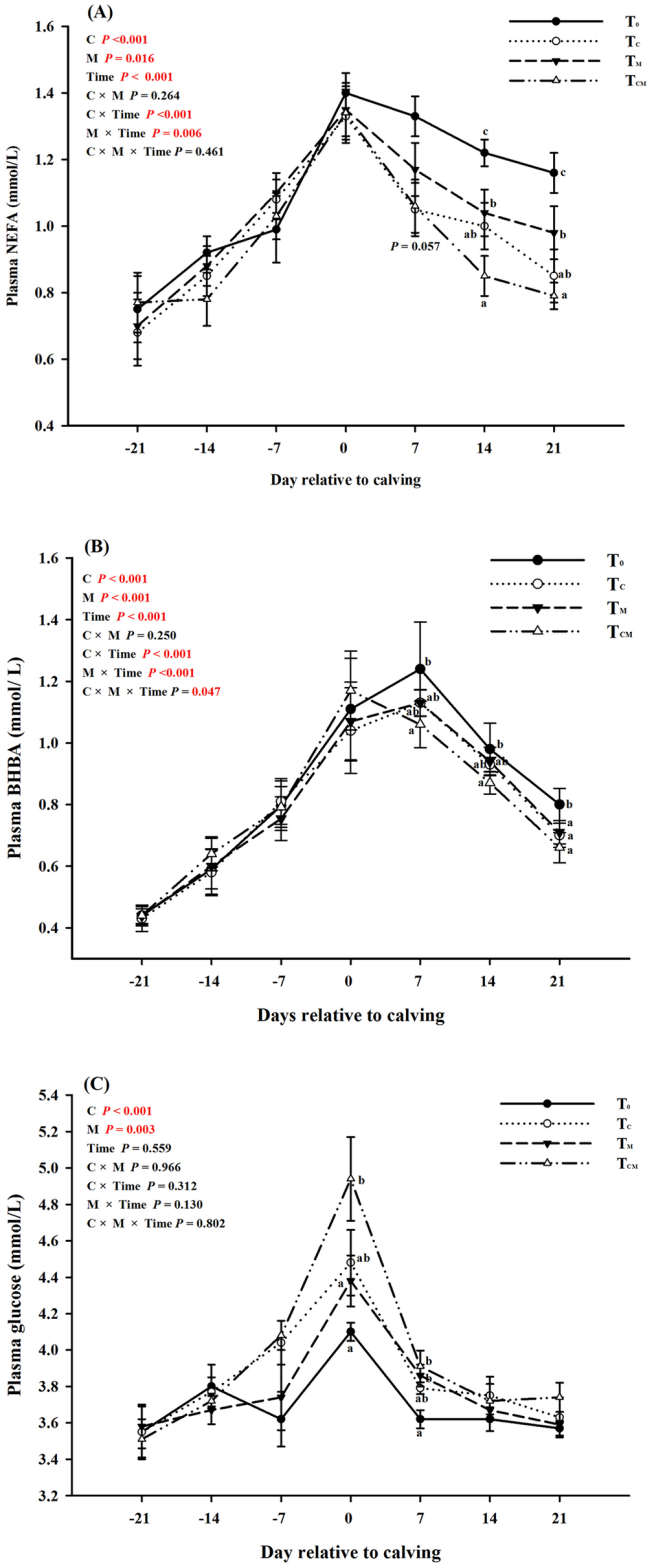
Dynamic effects of supplementation with rumen-protected choline (T_C_), rumen-protected methionine (T_M_) or both (T_CM_) on plasma levels of non-esterified fatty acids (A), β-hydroxybutyric acid (B) and glucose (C) in transition dairy cows. ^a, b, c^ Values with different superscript letters at the same time point are significantly different (*P* < 0.05).

The weekly plasma NEFA concentration continuously increased over the three weeks of the prepartum period and then declined progressively from calving day until day 21 postpartum (Fig 3A). Over the prepartal period, there was no significant difference in the NEFA concentration between the four groups (Table 2, *P* > 0.05). However, supplementation with RPC, RPM or both decreased the plasma NEFA concentration during the postpartal period (Table 2); the greatest decline in the plasma NEFA concentration was observed in the T_CM_ group, followed by the T_C_ group and the T_M_ group (Table 2 and Fig 3A). This decrement displayed statistical significance on days 14 and 21 postpartum (Fig 3A, *P* < 0.05). The effects of supplements on plasma BHBA levels were similar with that of NEFA (see Table 2 and Fig 3A). The highest BHBA level appeared at 7 d, which was different from NEFA (0 d).

As shown in Fig 3C, the weekly plasma glucose concentration displayed a quadratic pattern over the experimental period due to a sharp elevation on calving day in all four groups. During the prepartum period, no significant effect was found among the four groups (Fig 3C, *P* > 0.05). The T_CM_ group exhibited the highest glucose concentration on calving day, followed by the T_M_, T_C_, and control groups. After calving, the plasma glucose concentration in all animals progressively declined until day 21 postpartum. On day 7 after calving, the glucose concentrations in both the T_CM_ and T_M_ groups, but not in the T_C_ group (*P* > 0.05), were higher than those in the control group (*P* < 0.05). On days 14 and 21 postpartum, the glucose concentration did not differ between the four groups (*P* > 0.05). Taking different stages of the transition period into consideration (Table 2), the glucose concentration was improved by RPC according to the main effect (*P* < 0.05). In addition, glucose levels during caving day and postpartum period were higher in the treatment groups than that of control (main effect *P* < 0.05, Table 2). As a whole, both RPC and RPM decreased plasma concentrations of NEFA and BHBA, and elevated plasma glucose level (main effect *P* < 0.05, Fig 3). No interactive effect existed between RPC and RPM supplementation (interaction *P* > 0.05), while significant interactions between additives and weeks were found on their effects on NEFA and BHBA concentrations (interaction *P* < 0.05).

**Fig 4:**
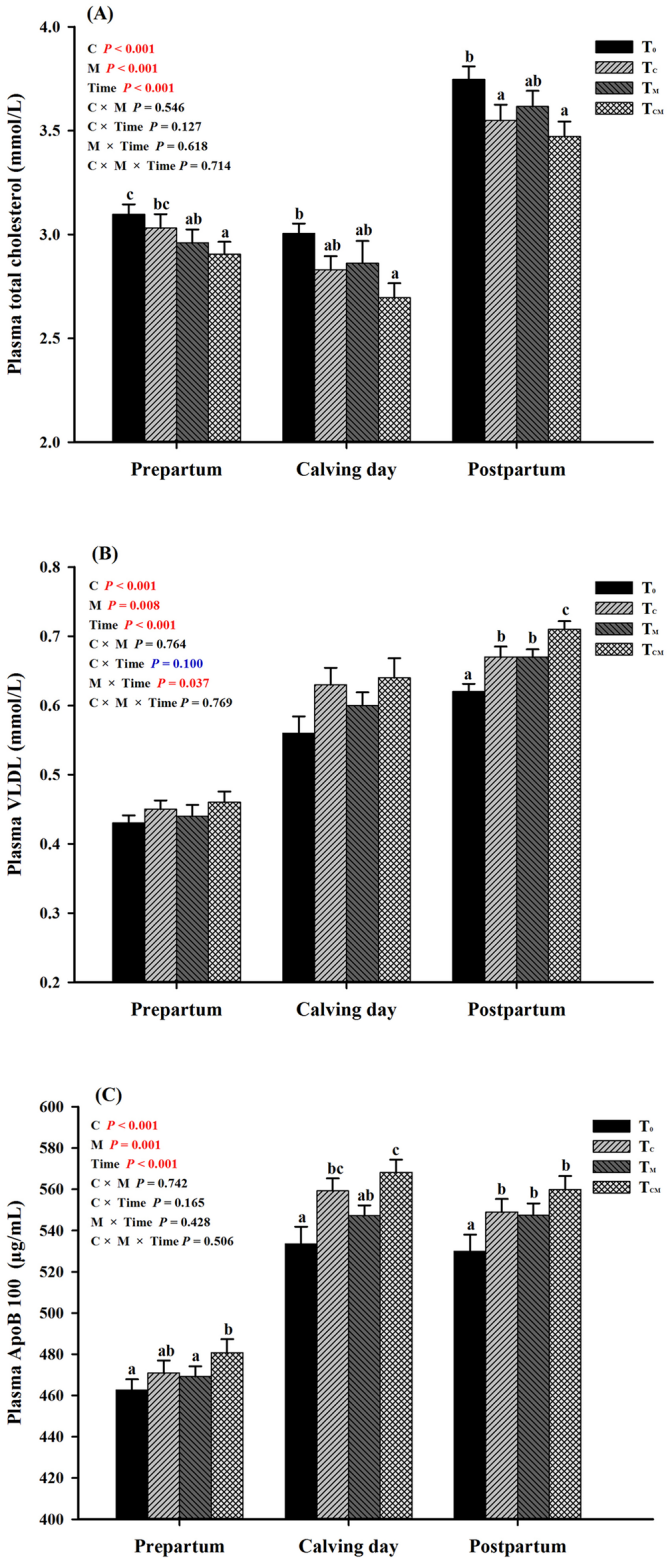
Plasma concentrations of total cholesterol (TC), very-low-density lipoprotein (VLDL) and apolipoprotein B100 (ApoB 100) in response to dietary supplementation of rumen-protected choline (T_C_), rumen-protected methionine (T_M_) or both (T_CM_) during periods of prepartum, calving day and postpartum in transition dairy cows. ^a, b, c^ Values with different superscript letters at the same time point are significantly different (*P* < 0.05).

The effects of supplementation with RPC and RPM on the mean levels of blood lipids over the six -week transition period were shown in Table 3. Supplementation with RPC, RPM or both significantly reduced the plasma concentrations of TC and LDL-C but increased the plasma contents of VLDL and ApoB 100 (*P* < 0.05). Supplementation with these nutrients did not significantly alter the plasma concentrations of TGs or HDL-C (*P* > 0.05), except that supplementation with RPC tended to elevate the HDL-C concentration (*P* = 0.082). The T_CM_ group had the highest ApoB 100 concentration and lowest TC level from prepartum to postpartum, while the postpartum VLDL content was increased by these two additives (*P* < 0.05, Fig 4). Values were significantly different in the six weeks (Time *P* < 0.001). The effect of RPM on plasma VLDL content showed an interaction with weeks (interaction *P* < 0.05), while RPC showed an interactive tendency with weeks (interaction *P* = 0.100).

**Table 3:** Effects of supplementation with rumen-protected choline (T_C_), rumen-protected methionine (T_M_) or both (T_CM_) on the plasma lipid concentrations (means of whole period) in transition dairy cows^1^.

Items	T_0_	T_C_	T_M_	T_CM_	SEM	*P*-value		
						C	M	C×M
TC, mmol/L	3.36	3.22	3.23	3.12	0.024	<0.001	<0.001	0.546
HDL-C, mmol/L	1.88	1.91	1.90	1.93	0.016	0.082	0.264	0.861
LDL-C, mmol/L	0.91	0.84	0.85	0.81	0.012	<0.001	<0.001	0.105
TG, mmol/L	0.14	0.13	0.14	0.13	0.007	0.296	0.550	0.834
VLDL, mmol/L	0.53	0.57	0.56	0.59	0.010	<0.001	0.008	0.764
ApoB 100, μg/mL	501.63	517.02	513.86	527.20	3.085	<0.001	0.001	0.742

^1^TC = total cholesterol, HDL-C = high-density lipoprotein cholesterol, LDL-C = low-density lipoprotein cholesterol, TG = triglyceride, VLDL = very-low-density lipoprotein, and ApoB 100 = apolipoprotein B100. Blood samples were collected at -21, -14, -7, 0, 7, 14, and 21 d.

### Biochemical metabolite levels in blood

**Fig 5:**
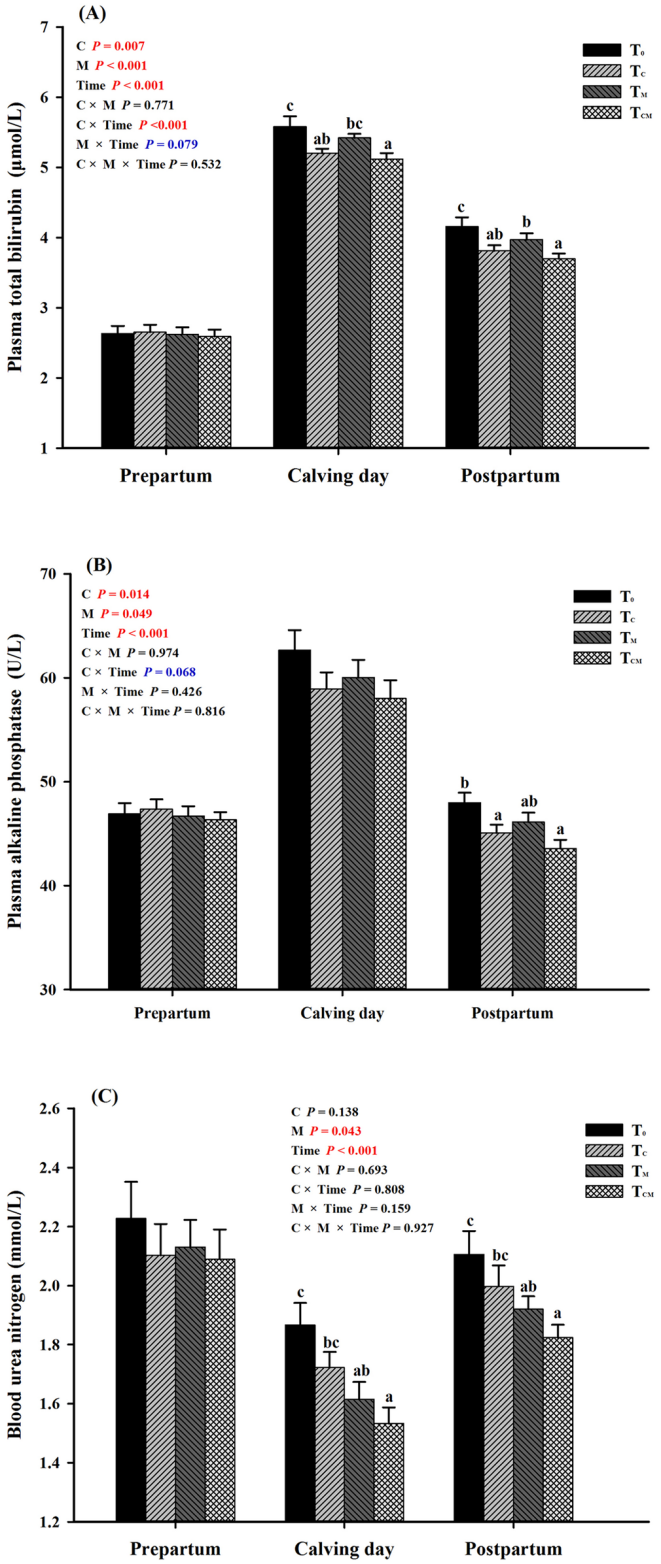
Plasma concentrations of total bilirubin (TBIL), alkaline phosphatase (ALP) and urea nitrogen in response to dietary supplementation of rumen-protected choline (T_C_), rumen-protected methionine (T_M_) or both (T_CM_) during periods of prepartum, calving day and postpartum in transition dairy cows. ^a, b, c^ Values with different superscript letters at the same time point are significantly different (*P* < 0.05).

No significant changes in the plasma concentrations of TP, ALB or GLB were detected in response to supplementation with RPC, RPM or both (*P* > 0.05, Table 4), except that RPC supplementation tended to increase the TP concentration (*P* = 0.083). Supplementation with RPC, RPM or both reduced the plasma TBIL concentration (*P* < 0.05). The activity of ALP in plasma was increased only in the T_CM_ group (*P* < 0.05). Supplementation with RPC, RPM or both did not significantly influence GPT activity (*P* > 0.05), and GOT activity in plasma was decreased only in the T_CM_ group compared with the control group (*P* < 0.05). Furthermore, supplementation with RPM tended to decrease the plasma BUN level (*P* < 0.05), whereas supplementation with RPC did not show such an effect (*P* > 0.05). After parturition, the combined supplements (T_CM_) decreased plasma TBIL concentration, ALP activity and BUN level (*P* < 0.05, Fig 5), while neither of them affected these parameters during the prepartum (*P* > 0.05), and strong time effects were detected (Time *P* < 0.001). In addition, the T_CM_ group presented the lowest contents of plasma TBIL and BUN in the calving day. Particularly, the RPM supplementation reduced BUN during the calving day and postpartum (*P* < 0.05, Fig 5C), but the T_C_ group showed no significant difference with control (*P* > 0.05, Fig 5C).

**Table 4:** Effects of supplementation with rumen-protected choline (T_C_), rumen-protected methionine (T_M_) or both (T_CM_) on the plasma levels of biochemical parameters (means of whole period) in transition dairy cows^1^.

Items	T_0_	T_C_	T_M_	T_CM_	SEM	*P*-value		
						C	M	C×M
TP, g/L	72.48	73.82	72.78	73.29	0.524	0.083	0.824	0.432
ALB, g/L	35.24	36.33	35.97	35.89	0.363	0.174	0.700	0.112
GLB, g/L	37.23	37.49	36.80	37.40	0.699	0.543	0.714	0.807
TBIL, μmol/L	3.70	3.51	3.60	3.43	0.034	<0.001	0.007	0.771
GPT, U/L	20.18	19.90	20.95	18.70	0.533	0.022	0.070	0.687
GOT, U/L	86.84	85.54	86.43	84.17	0.800	0.031	0.271	0.546
ALP, U/L	49.61	48.04	48.35	46.82	0.611	0.014	0.049	0.974
BUN, mmol/L	2.12	2.00	1.97	1.90	0.063	0.138	0.043	0.693

^1^TP = total protein, ALB = albumin, GLB = globulin, A/G = ALB/GLB, TBIL = total bilirubin, GPT = glutamate pyruvate transaminase, GOT = glutamic oxalacetic transaminase, ALP = alkaline phosphatase, and BUN = blood urea nitrogen. Blood samples were collected at -21, -14, -7, 0, 7, 14, and 21 d.

### Lactation performance

Changes of lactation performance in response to dietary RPC and RPM were presented in Table 5. Both RPC and RPM improved the 4% fat-corrected milk (FCM) production and the yield of total milk solids (*P* < 0.05). Supplementation with RPM increased percentages of milk protein and non-fat solids (*P* < 0.05), and tended to increase milk fat content (*P* = 0.071). The RPC improved milk fat content (*P* < 0.05) and showed a trend to increase non-fat solids (*P* = 0.064). No interaction between RPC and RPM was detected (interaction *P* > 0.05).

**Table 5:** Effects of dietary supplementation of rumen-protected choline (T_C_), rumen-protected methionine (T_M_) or both (T_CM_) on postpartum lactation performance in transition dairy cows^1^.

Items	T_0_	T_C_	T_M_	T_CM_	SEM	*P*-value		
						C	M	C×M
FCM (kg/d)	22.70	23.42	23.31	23.94	0.264	0.015	0.040	0.868
Milk fat (%)	3.28	3.44	3.41	3.60	0.078	0.027	0.071	0.855
Milk protein (%)	3.05	3.19	3.23	3.28	0.056	0.103	0.022	0.366
Milk lactose (%)	4.84	4.88	4.87	4.85	0.030	0.811	0.991	0.424
Total milk solids (%)	12.01	12.88	12.90	13.26	0.250	0.019	0.015	0.315
Somatic cell count (×10^4^/mL)	21.55	18.63	20.18	18.37	2.37	0.325	0.732	0.816
Non-fat solids (%)	8.73	9.43	9.49	9.66	0.228	0.064	0.036	0.245
Milk urea nitrogen (mg/dL)	11.20	10.31	10.86	10.40	0.576	0.245	0.827	0.710

^1^FCM = 4% fat-corrected milk.

### Plasma antioxidant status

**Fig 6:**
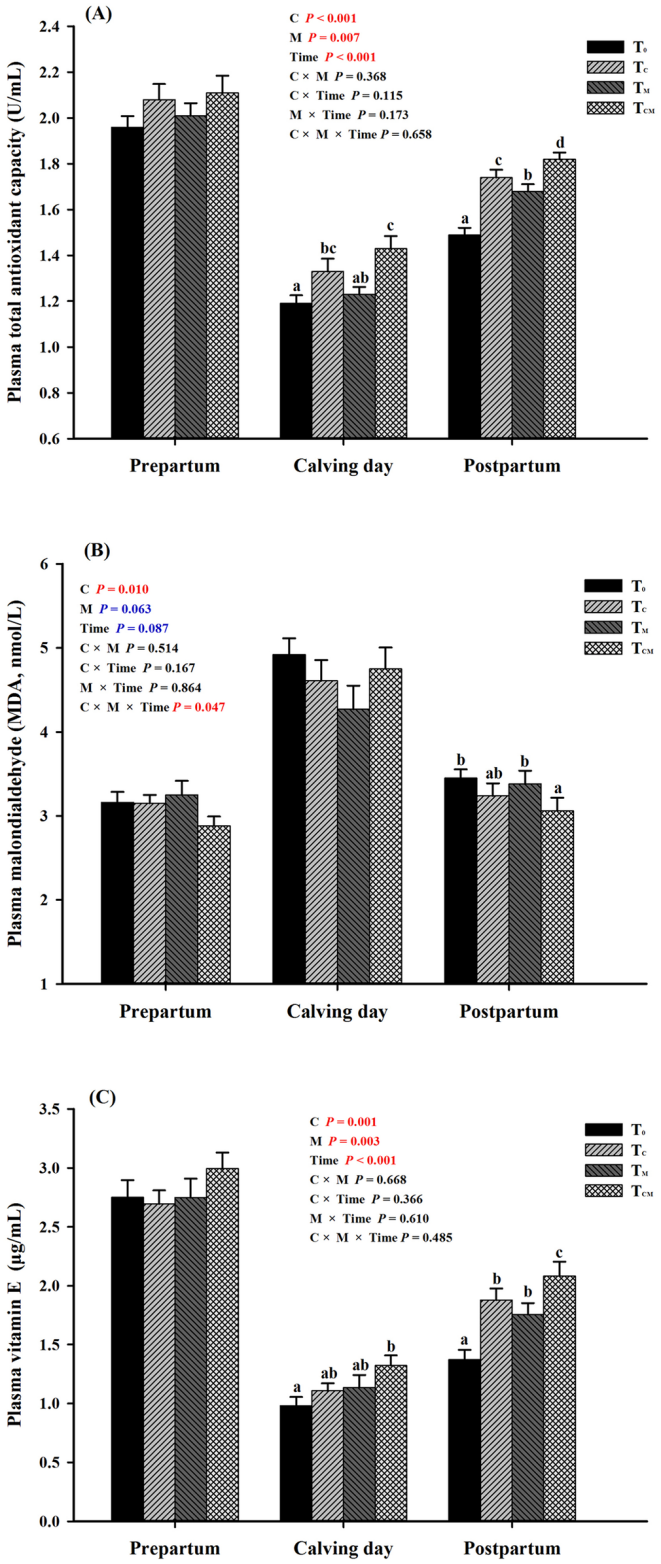
Plasma total antioxidant capacity (T-AOC) and levels of malonaldehyde (MDA) and vitamin E in response to dietary supplementation of rumen-protected choline (T_C_), rumen-protected methionine (T_M_) or both (T_CM_) during periods of prepartum, calving day and postpartum in transition dairy cows. ^a, b, c^ Values with different superscript letters at the same time point are significantly different (*P* < 0.05).

The levels of indicators of antioxidant status and capacity in plasma are shown in Table 6. The T-AOC, GSH-Px activity and the vitamin E concentration were increased by supplementation with RPC, RPM or both (*P* < 0.05). Supplementation with RPC significantly reduced the MDA concentration (*P* > 0.05), and supplementation with RPM tended to reduce the MDA concentration (*P* = 0.063). No supplementation treatment significant impacted SOD or CAT activity. The effects on plasma T-AOC, MDA and vitamin E were closely related to time factor (Fig 6, time *P* <0.001). In practical terms, the T-AOC in the T_C_ and T_CM_ groups were higher in comparison with T_0_ group at the calving day (*P* < 0.05), and the vitamin E level was higher than the control on the same day (*P* < 0.05). Both supplements enhanced plasma T-AOC and increased vitamin E concentration in the postpartum period (*P* < 0.05), and the peak value appeared in the T_CM_ group (Fig 6). As for the plasma MDA content, only the T_CM_ group presented a higher level than the control after parturition (*P* < 0.05). Any changes of these indexes were not discovered in the prepartal period (*P* > 0.05), and no interactive effect was found during the whole transition period either (*P* > 0.05).

**Table 6:** Effects of supplementation with rumen-protected choline (T_C_), rumen-protected methionine (T_M_) or both (T_CM_) on the plasma indicators of antioxidant status (means of whole period) in transition dairy cows^1^.

Items	T_0_	T_C_	T_M_	T_CM_	SEM	*P-*value		
						C	M	C×M
T-AOC, U/mL	1.65	1.83	1.77	1.89	0.031	<0.001	0.007	0.368
MDA, nmol/mL	3.54	3.40	3.45	3.23	0.068	0.010	0.063	0.514
GSH-Px, U	101.74	109.08	107.93	111.87	1.713	0.002	0.012	0.327
SOD, U/mL	12.60	13.00	13.16	13.29	0.294	0.367	0.153	0.656
CAT, U/mL	6.48	6.59	6.78	6.77	0.345	0.886	0.488	0.857
Vitamin E, μg/mL	1.91	2.12	2.09	2.37	0.070	0.001	0.003	0.668

^1^T-AOC = total antioxidant capacity, MDA = malondialdehyde, GSH-Px = glutathione peroxidase, SOD = superoxide dismutase, and CAT = catalase. Blood samples were collected at -21, -14, -7, 0, 7, 14, and 21 d.

### Immune responses

**Fig 7:**
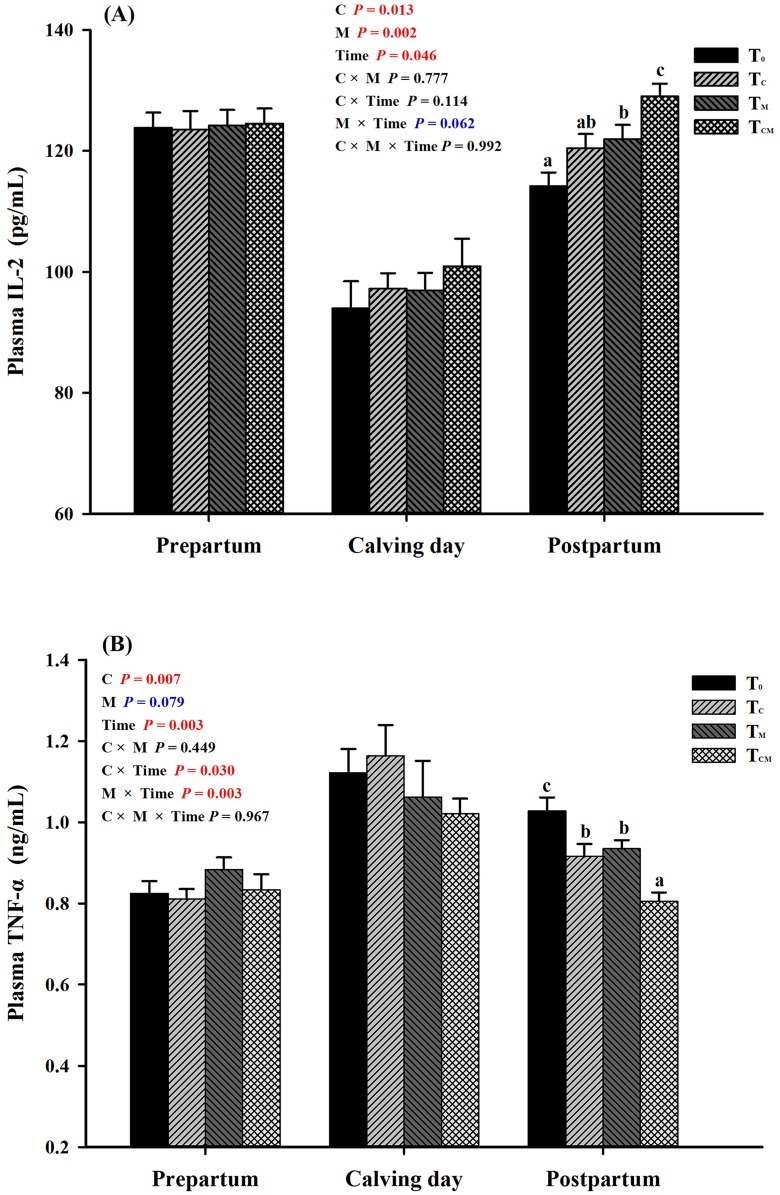
Plasma levels of interleukin 2 (IL2) and tumor necrosis factor α (TNF-α) in response to dietary supplementation of rumen-protected choline (T_C_), rumen-protected methionine (T_M_) or both (T_CM_) during periods of prepartum, calving day and postpartum in transition dairy cows. ^a, b, c^ Values with different superscript letters at the same time point are significantly different (*P* < 0.05).

The plasma levels of pro-inflammatory cytokines are shown in Table 7. Either RPC or RPM supplementation resulted in an increased IL-2 level and a decreased IL-6 level (*P* < 0.05). RPC supplementation reduced the TNF-α concentration (*P* < 0.05), and RPM supplementation tended to reduce the TNF-α concentration (*P* = 0.079). Neither RPC nor RPM significantly influenced the IL-4 level (*P* > 0.05). During the prepartal period and calving day, plasma concentrations of IL-2 and TNF-α were not affected by any of the additives (Fig 7, *P* > 0.05). After calving, compared with the control, there was higher IL-2 level in the T_M_ and T_CM_ group, and the T_CM_ group showed an enhanced concentration than the T_M_ group (*P* < 0.05). The plasma TNF-α content in all of the supplemented groups was increased (*P* < 0.05), and the maximum appeared in the T_CM_ group (Fig 7). The interactions between additives and time (C × Time and M × Time) were significant (interaction *P* < 0.05). In addition, the effect of RPM on plasma IL-2 level tended to be interactive with time (interaction *P* < 0.062).

**Table 7:** Effects of supplementation with rumen-protected choline (T_C_), rumen-protected methionine (T_M_) or both (T_CM_) on the blood levels of pro-inflammatory cytokines (means of whole period) in transition dairy cows (N = 7)^1^.

Items	T_0_	T_C_	T_M_	T_CM_	SEM	*P*-value		
						C	M	C×M
IL-2, pg/mL	115.47	118.48	119.35	123.08	1.256	0.013	0.002	0.777
IL-4, ng/mL	1.07	1.08	1.06	1.11	0.021	0.133	0.604	0.394
IL-6, pg/mL	165.53	150.41	153.40	136.72	4.336	0.001	0.007	0.859
TNF-α, ng/mL	0.96	0.91	0.93	0.85	0.022	0.007	0.079	0.449

^1^IL = interleukin, and TNF-α = tumor necrosis factor-α. Blood samples were collected at -21, -14, -7, 0, 7, 14, and 21 d.

**Fig 8:**
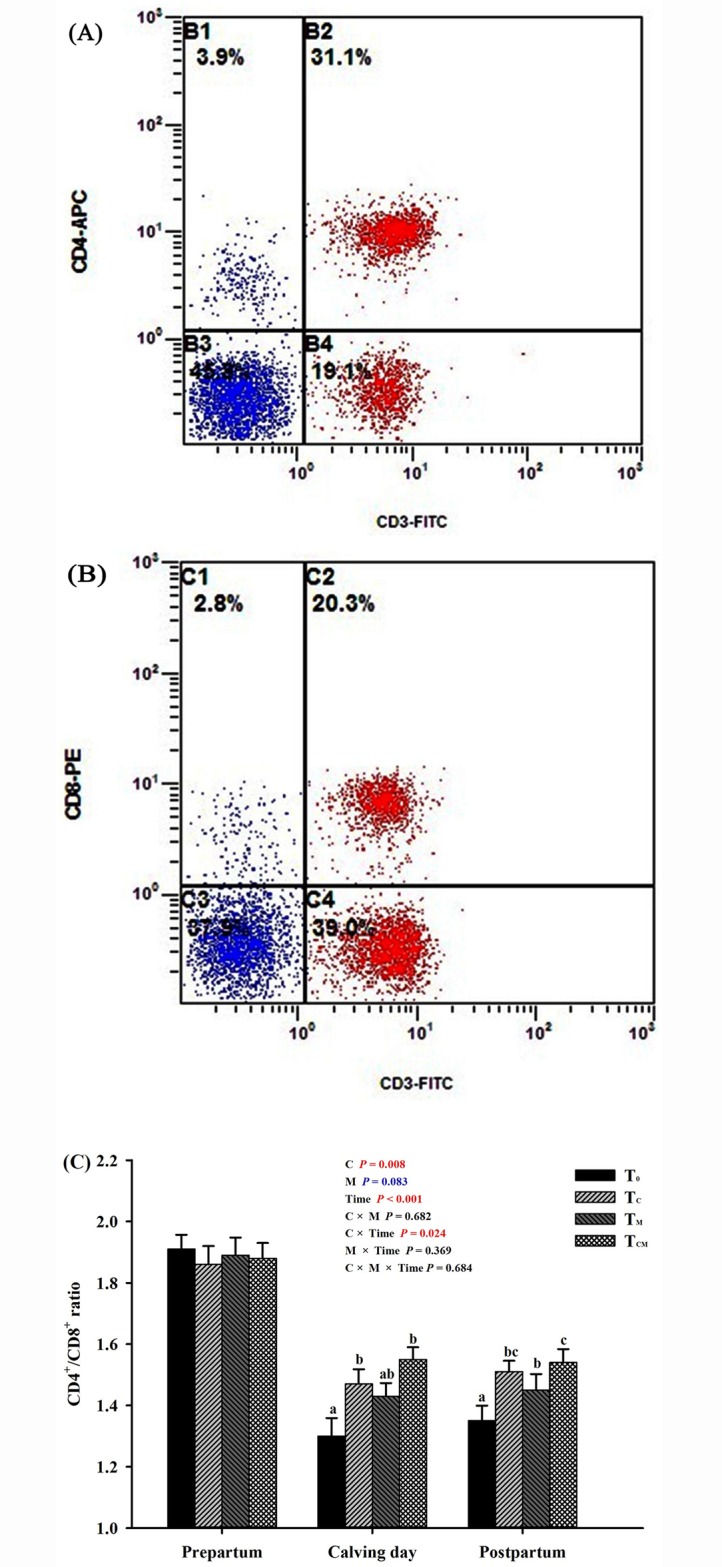
Effects of supplementation with rumen-protected choline (T_C_), rumen-protected methionine (T_M_) or both (T_CM_) on proportions of T lymphocyte subtypes in peripheral blood of transition dairy cows. (**A**) and (**B**) present the output images from flow cytometry. (**C**) displays the effects of each treatment over the three weeks before calving, on calving day, and over the three weeks after calving. ^a, b, c^ Values with different superscript letters at the same time point are significantly different (*P* < 0.05).

Data for the CD4^+^/CD8^+^ ratio were analyzed during the prepartal period, on calving day, and during the postpartal period, as well as over the entire transition period (Fig 8). No significant difference in the CD4^+^/CD8^+^ ratio was observed between the four groups over the three weeks before calving (*P* > 0.05). On calving day, the CD4^+^/CD8^+^ ratio was higher in the T_C_ and T_CM_ groups than that in the control group (*P* < 0.05). Over the three weeks after calving, the CD4^+^/CD8^+^ ratio was significantly higher in all three supplemented groups than the control group (*P* < 0.05). Considering the entire transition period, the T_C_ and T_CM_ groups, but not the T_M_ group (*P* > 0.05), displayed a higher CD4^+^/CD8^+^ ratio than the control group (*P* < 0.05).

## Discussion

It is well known that the metabolic characteristics of cows during the transition period are markedly different from those during other periods. Due to a dramatic DMI reduction and hormone-induced adaptations, cows typically experience serious imbalances in the levels of energy, amino acids and other nutrients [3,9]. A deficiency in energy intake results in intensive NEB-induced adipose fat mobilization and enhanced activity of lipid metabolic cycles in the liver of dairy cows. Alleviating NEB during the transition period is a top priority in dairy nutrition. Methionine is a limiting amino acid for milk production, and its supplementation could ameliorate amino acid imbalance. Choline and methionine can also alleviate the oxidative stress that occurs throughout the transition period [21–23]. Thus, the effects of supplementation of these nutrients on metabolism under NEB conditions were examined. In this experiment, the treated transition cows fully consumed 60.0 g/d ReaShure^®^ and 17.7 g/d Mepron^®^; these doses are within the suggested supplementation range [25,34].

In the present study, RPC and RPM enhanced postpartal DMI by greater than 1.0 kg/d, thereby increasing the dietary intakes of NE_L_ and MP. This result is in agreement with published data of transition cows [35,36]. Lima et al. [35] reported a tendency of increasing DMI due to RPC supplementation. Based on a meta-analysis, Patton [36] found that RPM supplementation enhanced feed intake. However, another report indicated that neither RPC nor RPM supplementation affected DMI in transition dairy cows [37]. These disparate results could be attributed to differences in the nutrient levels (energy, CP, etc.) and in the rumen bypass ratios of products. However, in this study these two additives did not influence DMI before calving. The cows were likely not under NEB conditions before calving [5], and this supposition is supported by the calculated energy balance as well as lower plasma concentrations of NEFA and BHBA during the prepartal period. In fact, these additives typically exert stronger regulatory effects under more intense NEB circumstances [9]. Another explanation of this discrepancy could be that the modulatory effects of choline and methionine were time-dependent; this phenomenon has been reported by Benefield et al. [38] and Lima et al. [9]. In summary, dietary supplementation with RPC and RPM could improve nutrient intake in postpartal dairy cows, which was in accordance with Osorio et al. [39] who found an increased postpartal DMI by a rumen bypass methionine product.

The RPC and RPM supplementation reduced the plasma levels of NEFA and BHBA in this study, demonstrating an alleviative NEB. This inference is directly evidenced by the elevated energy balance value calculated according to NRC (2001). To adapt to the special physiological state of nutrient deficit, transition cows utilize body deposits, predominantly consisting of lipids and, to a lesser extent, proteins [40,41]. A large quantity of NEFA are released from adipose tissue into the circulation and are then transported to the liver for further metabolism to produce energy. Subsequently, NEFA are completely oxidized to form CO_2_ and H_2_O, are incompletely oxidized to form ketone bodies (primarily BHBA), or are re-esterified to form TGs [11,42]. The hepatic accumulation of TGs and an increased plasma concentration of BHBA cause fatty liver and ketosis, resulting in impaired reproduction, reduced lactation performance and various metabolic disorders [43]. Therefore, the plasma concentrations of BHBA and NEFA have been considered as effective indicators of energy status in transition cows [44,45]. The decrease in the NEFA concentration caused by RPC supplementation observed in this study is in accordance with the findings of Pinotti et al. [34] and Cooke et al. [16]. Moreover, our finding of a reduced BHBA level due to RPC supplementation is supported by Elek et al. [17]. However, several studies reported no effect of RPC supplementation on these two indicators [10,14,46]. These discrepant results could be due to differences in basal diet, doses of additives and body condition scores. To date, very few experiments have focused on the influence of methionine on energy balance in transition cows. Durand et al. [47] reported reduced plasma levels of ketones during early lactation in high-yielding dairy cows, but neither RPC nor RPM influenced the NEFA or BHBA concentrations in the study presented by Ardalan et al. [15].

Methionine and choline metabolism are closely interrelated in ruminants, and both of these nutrients function as methyl moiety providers. Methionine acts as a methyl donor to promote *de novo* synthesis of choline, and choline supplementation has been demonstrated to spare methionine, a sulfur-containing amino acid considered as the first limiting amino acid for milk protein synthesis [15]. Supplementation of RPC and RPM improved milk yield and percentages of milk fat and protein, which was also observed in a RPM-supplemented research by Osorio et al [39]. As underscored in NRC (2001), sufficient MP and adequacy of methionine in MP are of great importance for lactation performance. Actually, methionine and choline are involved in analogous metabolic pathways and regulatory mechanisms. Choline facilitates the transfer of NEFA to the liver, particularly to hepatic mitochondria, by modulating the gene expression of fatty acid transport protein 5 (**FATP5**) and by increasing the availability of carnitine, an essential cofactor of fatty acid β-oxidation [48]. Specifically, choline promotes the accumulation of carnitine via two pathways: promoting carnitine synthesis in the liver and enhancing the incorporation of dietary carnitine into hepatocytes. Furthermore, carnitine palmitoyl transferase-1 (**CPT-1**), the transporter that regulates NEFA entry into mitochondria via the conversion of activated fatty acids into acylcarnitines, plays a crucial role in hepatic fatty acid β-oxidation [49,50]. Evidence has indicated that choline and methionine act on hepatic metabolism by regulating CPT-1 gene expression. Serviddio et al. [51] demonstrated that decreased CPT-1 expression impaired hepatic fatty acid β-oxidation in rats fed a choline- and methionine-deficient diet. A latest study by Osorio et al. [52] found that dietary methionine could function as a methyl donor to regulate hepatic lipid metabolism via the promoter methylation of some key regulators in transition cows, such as peroxisome proliferator-activated receptor alpha (PPARα). However, the epigenetic data is still insufficient, and more trials are needed.

In ruminant animals, glucose is derived via hepatic gluconeogenesis using volatile fatty acids (VFA, primary substrate), lactate and amino acids as substrates and via direct intestinal uptake. As a result, adequate glucose provision could decrease fat mobilization from adipose tissue for β-oxidation as well as spare glucogenic amino acids. Supplementation with RPC and RPM decreased the plasma cholesterol contents (TC and HDL-C) and increased the glucose level in this experiment. It is plausible that, as we stated, choline and methionine promote the hepatic complete oxidation of NEFA, and more acetyl-CoA, produced at least in part from mitochondrial β-oxidation, are oxidized to CO_2_ and H_2_O. Thereby, less acetyl-CoA, acted as substrate, are available to sustain cholesterol synthesis. Moreover, the increased concentrations of VLDL and ApoB 100 observed in this study suggested that both additives were advantageous to lipid transfer out of the liver, and this observation is in accordance with the finding of Goselink et al. [48]. These results suggested that fatty infiltration of the liver could be alleviated, thus enhancing liver function. It has been reported that fatty infiltration of the liver in transition cows restricts gluconeogenesis and other metabolic activities [53,54]. Goselink et al. [48] concluded that dietary RPC supplementation enhanced hepatic carbohydrate and energy metabolism of transition cows. It could be plausibly speculated that hepatic gluconeogenesis was accelerated in accordance with increased postpartum gene expression of glucose transporter 2 (**GLUT2**). As a consequence, the improvement in carbohydrate metabolism alleviated the NEB and, in turn, mitigated body fat mobilization. The feedback mechanism involved in the mobilization of fatty acids to maintain energy balance remains unclear. In particular, dietary RPC and RPM supplementation could ameliorate NEB in transition dairy cows by three potential mechanisms: (1) promoting hepatic β-oxidation of NEFA; (2) facilitating VLDL synthesis to transport excess TGs, thereby reducing TG deposition in the liver; and (3) elevating the circulating glucose levels by enhancing gluconeogenesis in the liver.

The plasma TBIL concentration is an important indicator of liver function that is increased during severe lipidosis [55–57]. A decrease in the plasma TBIL concentration could indicate a healthy liver status and improvement of hepatic function in transition cows receiving RPC and RPM supplementation in this experiment. The reduction in the TBIL level could be a result of accelerated NEFA β-oxidation and TG transport. This conclusion was supported by the reduced plasma activities ALP and GPT, two hepatic intracellular enzymes that reflect liver status. In practical terms, the abnormal release of these enzymes into the bloodstream usually indicated liver damage, such as lysis and necrosis [39]. Consequently, changes of these parameters in the supplemented groups indicated an improvement of liver function, especially for the postpartum cows.

Notably, supplementation with RPM, but not RPC, reduced the plasma BUN level, and this result suggested that RPM supplementation promoted nitrogen utilization in dairy cows. The BUN level is typically used to estimate nitrogen excretion and nitrogen utilization efficiency in animals [58]. Choline, which is classified as a B-complex vitamin, is proposed to preferentially regulate energy metabolism in transition dairy cattle. Zom et al. [10] described that choline functions as a methyl donor to spare methionine, ultimately increasing the use of methionine for protein synthesis. As a consequence, the regulation of nitrogen metabolism by choline appears to be an indirect process, which needs further study. Methionine directly modulates protein synthesis via the mammalian target of rapamycin (mTOR) and Janus kinase 2 (JAK2)-signal transducer and activator of transcription 5 (STAT5) signaling pathways [59].

In this experiment, as a result of supplementation with RPC and RPM, the plasma T-AOC, GSH-Px activity and vitamin E concentration were increased, whereas the MDA content was reduced; these alterations manifested as an enhancement in antioxidant capacity. The production and breakdown of free radicals remains in homeostasis in dairy cows. During the transition period, increased levels of reactive oxygen species (**ROS**) are generated because of intensive hepatic NEFA oxidation after body fat mobilization, and this increase in ROS production results in the development of oxidative stress [4,55]. The antioxidant barrier in animals consists of the antioxidase system (GSH-Px, CAT, SOD, etc.) and non-enzymatic antioxidants (vitamin E, vitamin C, glutathione, etc.). Methionine has been evidenced to promote GSH-Px synthesis in bovine mammary epithelial cells [60]. Besides, supplemental RPM has been proved to alter hepatic gene expressions in the methionine cycle of transition cows, boosting productions of antioxidants such as glutathione and taurine [61]. Therefore, methionine plays a vital role in maintaining the antioxidant status of transition cows. Vitamin E is a lipid-soluble vitamin, and VLDL accompanies vitamin E during its incorporation and transport [62]. Thus, enhancing VLDL synthesis increases the circulating vitamin E level. Similarly, paraoxonase-1 (**PON1**), a significant component of the mammalian natural antioxidant system, is bound to high-density lipoprotein (**HDL**). For this reason, the trend of an elevation in the HDL-C levels by RPC supplementation might increase the availability of PON1, but this parameter was not evaluated in this study. Alternatively, choline has been demonstrated to function as a homeostatic factor by regulating the antioxidant response in fish via the nuclear factor erythroid 2 (NF-E2)-related factor 2 (Nrf2) signaling pathway [63], although this role of choline has yet to be studied in dairy cows. Thus, it could be deduced that the alleviation of NEB by RPM and RPC reduces the production of NEFA, thereby decreasing the uptake of hepatic NEFA and, thus, the generation of ROS.

Elevated levels of NEFA and ketone bodies in transition dairy cows are the principal stimulators of immunosuppression, inflammatory responses and metabolic disorders [1]. There are two important subsets of T lymphocytes, CD4^+^ and CD8^+^ T cells, and their relative quantity (CD4^+^/CD8^+^ ratio) in peripheral blood is widely accepted as an indicator of immune status [30]. Specifically, CD4^+^ T cells are primarily composed of T-helper 1 (Th1) and Th2 cells [64,65]. The IL-2, a T-cell growth factor involved in T-cell proliferation, is secreted by Th1 cells, whereas IL-4 and IL-6 are secreted by Th2 cells [64,65]. The IL-4 can promote the proliferation and differentiation of B lymphocytes as well as the secretion of antibodies [32]. The IL-6 modulates the immune response to the initiation of detrimental infection and damage, and TNF-α, a pro-inflammatory cytokine derived from macrophages and monocytes, performs similar functions [66]. In this experiment, the increased plasma IL-2 level and CD4^+^/CD8^+^ ratio in peripheral blood as well as the reduced plasma concentrations of IL-6 and TNF-α indicated that RPC and RPM may improve the immune function of transition cows. First, the impaired functions of immune cells are partly recovered due to the attenuation of NEB [9]. Second, the enhanced antioxidant capacity may alleviate oxidative damage to immune cells. Wu et al. [23] first demonstrated that choline directly modulates immune function via the mTOR pathway in fish. Whether this activity also occurs in dairy animals requires further experimentation. In addition, as indicated by better blood leukocyte-killing capacity, Osorio et al. [39] found improved immune function in RPM-supplemented postpartum cows. To authors’ knowledge, this is the first study where the CD4^+^/CD8^+^ ratio of T cell subtypes is influenced by dietary RPC and RPM supplementation, providing new corroborative evidence for their immunological enhancement in transition cows.

Overall, there were no interactions between RPC and RPM with respect to the plasma levels of metabolites and body health biomarkers in transition dairy cows examined in this study, but the interactive effects between treatments and time were discovered in some parameters. To our knowledge, interaction effects between RPC and RPM in transition cows have rarely been examined, specifically in only one study by Ardalan et al. [15], who detected no such interaction. Therefore, the RPC and RPM appear to independently modulate the metabolism of transition dairy cows in this study, and the time-dependent effects should be considered in dairy production.

## Conclusions

Dietary supplementation with RPM and RPC alleviated negative energy balance by stimulating postpartal feed intake and modulating hepatic lipid metabolism, and both of these additives improved the postpartum lactation performance as well as the health (antioxidant status and immune function) of transition dairy cows. However, the detailed molecular network, including the epigenetic mechanism, remains to be established, and omics technology might be an effective strategy to further understand these mechanisms.
